# Healthcare-associated (HA) bloodstream infections (BSI) secondary top surgical site infections: surveillance program across Quebec hospitals (2007 to 2010)

**DOI:** 10.1186/1753-6561-5-S6-P63

**Published:** 2011-06-29

**Authors:** V Blouin, E Fortin, I Rocher, A Fortin, C Tremblay, C Frenette, C Quach, SPIN Network

**Affiliations:** 1 McGill University, Montreal, Canada; 2 INSPQ, Quebec, Canada; 3 INSPQ, Montreal, Canada; 4 CHUQ, Quebec, Canada

## Introduction / objectives

To describe HA-BSI secondary to surgical site infections (SSI) in Quebec hospitals.

## Methods

Acute care centers with ≥ 1000 admissions/year were invited to participate voluntarily and declare via a web portal all HA-BSIs secondary to a SSI between 2007 and 2010.The surveillance program also included outpatients and paediatric patients.

## Results

56 of the 89 eligible centers participated (67% of short-term hospital beds), 64 % of those beds were in teaching hospitals. Of the 7709 episodes of HA-BSIs, 874 (11 %) were secondary to a SSI. Gastrointestinal surgeries were, by far, the leading cause of BSIs (244 – 28%) followed by cardiac surgery (132 – 15%), and orthopaedic surgery (98 – 11%). Implants were present in 25 cases (19%). The majority of SSIs (496 -57%) were organ space infections. S. aureus was the organism most often isolated in 28% (274), followed by E. coli in 13% (130) of patients. Overall, 79% of patients were admitted on a general/specialised ward upon onset, compared to 15% in ICU, and 6% in ambulatory care. The mean time to infection was 21 days with a median of 10 days (median time to infection for cardiac compared to gastrointestinal surgery: 11 and 8 days respectively). 75% of BSIs occurred in teaching hospitals.

## Conclusion

The Quebec surveillance program for HA-BSIs is a convenient way to survey the most severe SSIs, awaiting the development of a complete SSI surveillance program. Further analyses are needed to better understand the correlation between this targeted program and a complete SSI surveillance program.

## Disclosure of interest

None declared.

